# Distinct cellular dynamics following CD19 and CD7 CAR-T therapies revealed by high-dimensional CyTOF analysis and impact on clinical outcomes

**DOI:** 10.1515/jtim-2026-0081

**Published:** 2026-10-23

**Authors:** Zhipeng Zhou, Yiyang Ding, Zhuojun Liu, Zhifan Zhao, Ting Zhao, Xiaohui Zhang, Xiaojun Huang, Meng Lyu, Xuying Pei, Xiangyu Zhao

**Affiliations:** Peking University People's Hospital, Peking University Institute of Hematology, National Clinical Research Center for Hematologic Disease, Beijing Key Laboratory of Cell and Gene Therapy for Hematologic Malignancies, Beijing, China; Peking-Tsinghua Center for Life Sciences, Beijing, China

**Keywords:** CAR-T cell therapy, dynamic, immune reconstitution, cytometry by time-of-flight (CyTOF), non-relapse mortality

## Abstract

**Background and Objectives:**

CD7 and CD19 chimeric antigen receptor T-cell (CAR-T) therapies induce high remission in T-cell acute lymphoblastic leukemia; however, clinical outcomes of CD7 CAR-T therapy are less favorable compared with those of CD19 CAR-T therapy due to profound immune dysfunction, elevated infection, and non-relapse mortality (NRM). Whether these differences arise from distinct immune remodeling patterns that shape CAR-T cell persistence, functional fitness, and host immune recovery, remains unknown.

**Methods:**

We analyzed 74 patients (52 CD19; 22 CD7) who received CAR-T therapy. A subset of 28 patients underwent prospective immune monitoring with 107 longitudinal samples profiled using 40-marker cytometry by time-of-flight to assess CAR-T cell kinetics, exhaustion, host immune remodeling, and clinical associations.

**Results:**

High-dimensional profiling revealed strikingly divergent immune trajectories of CD19 and CD7 CAR-T therapies. At day 28, CD7 CAR-T recipients exhibited diminished CD8**^+^** CAR-T cell frequencies, early CAR-T cell exhaustion, and poor immune reconstitution, features more frequent among patients experiencing NRM. In contrast, CD19 CAR-T recipients showed sustained activation, low exhaustion, and prolonged persistence. These immunologic disparities translated into clinically meaningful differences where day-28 responses remained comparable (88.5% *vs*. 90.9%); however, CD7 CAR-T recipients developed higher late hematotoxicity and fatal infections, resulting in greater 1-year NRM (32.4% *vs*. 9.2%) and inferior overall survival (44.6% *vs*. 78.8%).

**Conclusion:**

CD7 and CD19 CAR-T therapies are associated with distinct patterns of immune remodeling and clinical outcomes. Compared with CD19 CAR-T therapy, CD7 CAR-T therapy was characterized by reduced CD8+ CAR-T persistence, early exhaustion, and delayed immune reconstitution, correlating with increased infections and higher non-relapse mortality.

## Introduction

Chimeric antigen receptor T-cell (CAR-T) therapy has transformed the treatment of relapsed or refractory hematologic malignancies.^[1, 2, 3]^ Among the available products, CD19^-^directed CAR-T cells are the most successful, particularly in B-cell acute lymphoblastic leukemia (B-ALL), with high remission rates and durable disease control.^[4,5]^ Recently, CD7^-^directed CAR-T therapy has emerged as a promising strategy for T-cell acute lymphoblastic leukemia (T-ALL), a disease with historically limited treatment options, and has demonstrated robust initial responses.^[6, 7, 8]^

Although both therapies induce high remission rates, evidence suggests that CD19 and CD7 CAR-T therapies trigger fundamentally distinct patterns of post-infusion immune remodeling. CD19 CAR-T therapy primarily disrupts the B-cell compartment and preserves global T-cell immunity, thus the longterm immune dysfunction is often managed with immunoglobulin replacement.^[9]^ In contrast, CD7 CAR-T therapy eliminates malignant cells alongside normal T cells, resulting in sustained T-cell aplasia, profound cytopenia, and heightened susceptibility to severe infections.^[10, 11, 12]^ These lineage-specific immunological consequences are key determinants of clinical outcomes, particularly the markedly elevated non-relapse mortality (NRM) observed in CD7 CAR-T recipients.^[13,14]^ Thus, while CD7 CAR-T therapy presents potent antileukemic activity, it also creates a unique and far more vulnerable immune environment.

Prior comparisons across different CAR-T constructs, such as CD19 CAR-T (axi-cel and tisa-cel) or BCMA CAR-T (cilta-cel and ide-cel), have shown that variations in signaling domains and manufacturing can shape the expansion, persistence, and toxicity profiles.^[15, 16, 17, 18]^ However, direct comparisons between CD19 and CD7 CAR-T therapies, which target distinct immune lineages rather than alternative forms of the same lineage, remain scarce. To date, only Zhang *et al*. evaluated the infection rates and hematologic toxicity of CD7 and CD19 CAR-T recipients.^[19]^ While this work provides valuable early insights, it offers limited data to characterize immune-cell functional states and lineage remodeling during immune reconstitution. Consequently, the mechanistic basis underlying divergent immune recovery patterns and their clinical implications remain elusive.

Given that CD 19 CAR-T remodels the B-cell niche,^[20,21]^ whereas CD7 CAR-T disrupts the T-cell lineage, their post-infusion immunological consequences are expected to differ substantially.^[22]^ However, current evidence is largely descriptive, and post-infusion immune remodeling remains poorly defined. Therefore, a high-dimensional profiling is essential to capture the distinct cellular dynamics following CD19 and CD7 CAR-T therapy and to evaluate their clinical impact.^[23]^

In this study, we systematically compared patients receiving CD19 or CD7 CAR-T therapy by integrating high-dimensional cytometry by time-of-flight (CyTOF) with clinical outcome data. Using a 40-marker CyTOF panel, we longitudinally mapped CAR-T cell dynamics, phenotypic and functional states, and endogenous immune reconstitution trajectories across major lymphoid and myeloid lineages. This study aimed to (i) delineate the distinct immune reconstitution landscapes following CD 19 and CD7 CAR-T cell infusion, and (ii) determine the role of these immune trajectories in shaping treatment durability, infection burden, and non-relapse mortality. Our study presents the first integrated, high-resolution immune atlas comparing CD19 and CD7 CAR-T therapies, revealing lineage-specific immune tradeoffs with direct implications for toxicity mitigation and clinical management.

## Methods

### Study participants and samples collection

Between July 2022 and July 2025, patients with relapsed or refractory (R/R) B-ALL or T-ALL/LBL who received CD19 or CD7 CAR-T therapy at Peking University People’s Hospital were screened for inclusion. Patients with follow-up shorter than 28 days or with incomplete laboratory data were excluded. Clinical data including patients baseline characteristics, previous therapies, clinical complications and last-follow-up were obtained. For detailed immune profiling, a consecutive cohort of 28 patients (18 CD19 CAR-T and 10 CD7 CAR-T recipients) treated between October 2024 and May 2025 was prospectively enrolled and analyzed. Peripheral blood samples were obtained at standardized timepoints: baseline (prior to fludarabine/ cyclophosphamide conditioning), day 4, 7, 10, 14, day 28, 2 months, and 3 months after infusion. The study protocol was approved by the Ethics Committee of Peking University People’s Hospital (Approval Nos. 2023PHD022–001, 2021PHC030–001, 2024PHC009–001, and 2024PHD020–001) and was conducted in accordance with the Declaration of Helsinki. Written informed consent was obtained from all participants or their legal guardians.

### Cytometry and analysis

Flow cytometry incorporating antibodies specific for the FMC63 scFv (CD19 CAR-T) or variable domain of heavy-chain-only antibody (VHH) domain (CD7 CAR-T) was used for sequential monitoring of circulating CAR-T cells. CyTOF was performed for high-dimensional profiling. Whole-blood samples underwent red blood cell lysis with ammonium-chloride-potassium (ACK) buffer and were sequentially stained with viability dyes, surface markers, and nucleic acid dyes, followed by fixation, permeabilization, and intracellular staining. A 40-marker CyTOF antibody panel (Supplementary Table S1) was designed to capture major immune lineages and functional programs, including activation, exhaustion, differentiation, chemotaxis, senescence, and immunomodulation. Data were acquired using a Helios mass cytometer (Fluidigm, USA) with EQ bead–based normalization.

Raw flow cytometry standard (FCS) files were processed using FlowJo v10.9.0 (FlowJo LLC, USA). Signal intensities were arcsinh-transformed, and unsupervised clustering was performed using Phenograph. Cell populations were annotated based on canonical marker expression and validated through manual gating. Dimensionality reduction was performed using t-distributed stochastic neighbor embedding (t-SNE) and uniform manifold approximation and projection (UMAP). Visualization and downstream analyses were conducted using ggplot2 and CATALYST in R.

### Antigen-specific stimulation assays

To evaluate antigen-induced functional responses, CAR-T cells were stimulated *in vitro* with viral peptide pools. Cytomegalovirus (CMV) pp65, CMV IE-1 and EBV EBNA1 and EBV LMP2a peptides (Miltenyi Biotec, Germany) were used at a final concentration of 1 μg/mL. Cells were incubated for 6 h at 37 °C in the presence of brefeldin A (BD) to enable intracellular cytokine accumulation. As a positive control, cells were stimulated with phorbol 12-myristate 13-acetate (PMA, 50 μg/L) and ionomycin (1 μg/mL).

### In vitro repetitive target-cell stimulation assay

To model chronic antigen exposure, CD7 and CD19 CAR-T cells were co-cultured with respective target cells (Jurkat for CD7, Raji for CD19) at a 1: 1 E: T ratio in culture medium supplemented with 100 U/mL IL-2, and restimulated with fresh targets every 3 days for 4 rounds. Post-stimulation, cells were harvested to compare bulk CD7 and CD19 CAR-T cells, and to evaluate the CD8^+^CD38^+^HLA^-^DR^+^ and CD8^+^CD38^⁻^HLA-DR^⁻^ subsets within the CD7 cohort. Cells were stained for surface markers (CD8, CD38, HLA-DR, PD-1, TIGIT, TIM-3), fixed/permeabilized (BD Cytofix/Cytoperm), and stained intracellularly for Perforin and Granzyme B. Data acquisition and analysis were performed using a Cytek Aurora cytometer and FlowJo v10.9.0 (FlowJo LLC, USA) software.

### Definition and evaluation criterion

Cytokine release syndrome (CRS) and classical immune effector cell-associated neurotoxicity syndrome (ICANS) were graded according to the American Society for Transplantation and Cellular Therapy (ASTCT) consensus criteria, with toxicity management following institutional guidelines.^[24]^ ICAHT was graded according to the European Hematology Association (EHA) and European Society for Blood and Marrow Transplantation (EBMT) consensus guidelines.^[25]^ Infections were defined as bacterial, viral, or fungal based on clinical syndromes combined with microbiologic confirmation, which included positive site/ blood cultures, polymerase chain reaction (PCR) assays, or specific serological antigen tests. Infection severity was classified as mild, moderate, severe, life-threatening, or fatal, according to previously published definitions. Specifically, mild infections required no treatment; moderate infections required oral treatment only (with the exception that bacteremia due to possible skin contaminants and fever without systemic symptoms were also categorized as moderate); severe infections required intravenous (IV) antimicrobial therapy or were associated with other severe clinical circumstances; life-threatening infections were complicated by life-threatening symptoms; and fatal infections were those that contributed significantly to patient death.^[26, 27, 28]^

### Statistical analysis

Continuous variables were compared using the Mann-Whitney *U* test or Wilcoxon signed-rank test, as appropriate. Categorical variables were analyzed using the chi-squared or Fisher’s exact test. Correlation analyses were performed using Spearman’s rank correlation coefficient, with correlation matrices visualized using the corrplot and ggplot2 packages in R. Time-to-event outcomes were evaluated using Kaplan–Meier survival curves with log-rank tests. Cox proportional hazards models were used to identify predictors of clinical outcomes. Statistical analyses were conducted using GraphPad Prism v10.0 (GraphPad Software, USA) and R v4.4.2. A two-tailed *P* value < 0.05 was considered statistically significant.

## Results

### Patient baseline characteristics

A total of 74 patients were enrolled, including 52 treated with CD19 CAR-T and 22 with CD7 CAR-T therapy. Baseline demographic and clinical characteristics are summarized in Table 1. The median follow-up duration was 10 months (range: 3–36 months). The two groups were comparable in gender, age, baseline leukemia burden, and history of allogeneic hematopoietic stem cell transplantation (Table 1).

**Table 1:** Baseline characteristics of the total 74 study population receiving CAR-T therapy

Characteristic	Total (*N* = 74)	CD19 CAR-T (*N* = 52)	CD7 CAR-T (*N* = 22)	*P*-value
Gender, *n* (%)				0.572
Male	40 (54.1)	27 (51.9)	13 (59.1)	
Female	34 (45.9)	25 (48.1)	9 (40.9)	
Age				0.481
Median (years, interquartile range)	32 (21, 41)	31 (21, 41)	33 (24, 39)	
Disease, *n* (%)				< 0.001
B-cell acute lymphoblastic leukemia	52 (70.3)	52 (100)	0 (0)	
T-cell acute lymphoblastic leukemia	22 (29.7)	0 (0)	22 (100)	
Bone marrow blast percentage, *n* (%)				0.093
<30%	36 (48.6)	22 (42.3)	14 (63.6)	
≥30%	38 (51.4)	30 (57.7)	8 (36.4)	
Pre_hematopoietic stem cell transplantation, *n* (%)				0.054
Yes	52 (70.3)	40 (76.9)	12 (54.5)	
No	22 (29.7)	12 (23.1)	10 (45.5)	
Response rate at day 28				> 0.999
Complete response/Partial response	66 (89.2)	46 (88.5)	20 (90.9)	
No response	8 (10.8)	6 (11.5)	2 (9.1)	
Cytokine release syndrome, *n* (%)				0.365
0	12 (16.2)	11 (21.2)	1 (4.5)	
1	20 (27)	13 (25)	7 (31.8)	
2	36 (48.6)	24 (46.2)	12 (54.5)	
3	6 (8.1)	4 (7.7)	2 (9.1)	
Immune effector cell–associated neurotoxicity syndrome, *n* (%)				0.72
0	61 (82.4)	41 (78.8)	20 (90.9)	
1	4 (5.4)	3 (5.8)	1 (4.5)	
2	5 (6.8)	4 (7.7)	1 (4.5)	
3	4 (5.4)	4 (7.7)	0 (0)	
Early immune effector cell–associated hematotoxicity, *n* (%)				0.523
0-2	23 (31.1)	15 (28.8)	8 (36.4)	
3-4	51 (68.9)	37 (71.2)	14 (63.6)	
Late immune effector cell–associated hematotoxicity, *n* (%)				0.023
0-2	45 (60.8)	36 (69.2)	9 (40.9)	
3-4	29 (39.2)	16 (30.8)	13 (59.1)	
Infection severity, *n* (%)				0.009
No	43 (58.1)	31 (59.6)	12 (54.5)	
Mild to severe	21 (28.4)	18 (34.6)	3 (13.6)	
Life-threatening to fatal	10 (13.5)	3 (5.8)	7 (31.8)	

CAR-T: chimeric antigen receptor T-cell; CD19 CAR-T: CD19-directed CAR-T therapy; CD7 CAR-T: CD7-directed CAR-T therapy.

To investigate immune reconstitution, a prospective subset of 28 consecutive patients underwent high-dimensional longitudinal immune monitoring (18 CD19 CAR-T and 10 CD7 CAR-T). Baseline demographic and disease characteristics were comparable across the two treatment cohorts, as summarized in Table 2. In total, 107 peripheral blood samples were collected, comprising 67 from the CD19 CAR-T cohort and 40 from the CD7 CAR-T cohort. Samples were obtained at five predefined timepoints: pre-infusion baseline (*n* = 28), day 14 (*n* = 26), day 28 (*n* = 28), month 2 (*n* = 15), and month 3 (*n* = 10) (Figure 1A and B).

**Figure 1:**
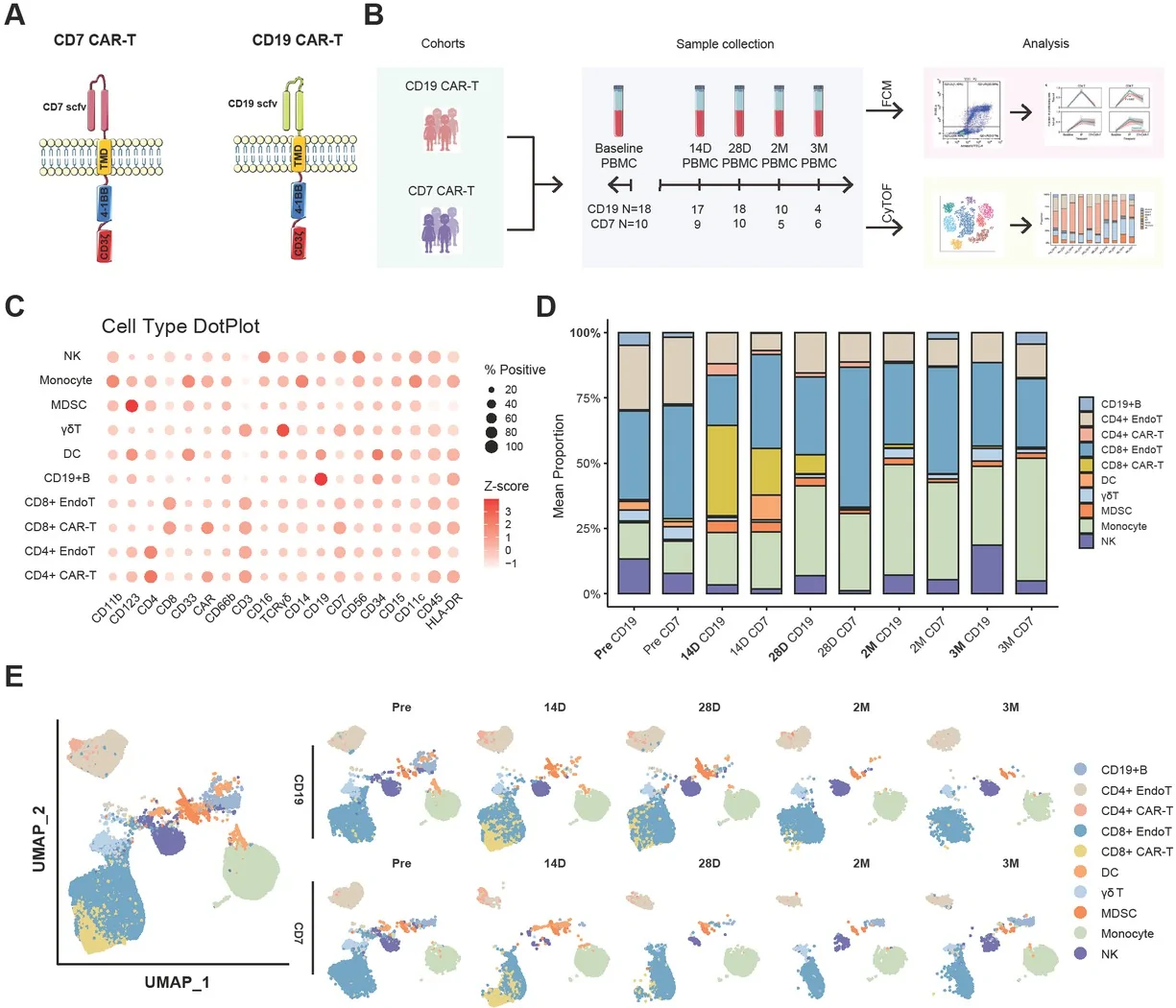
Study design, CAR constructs, and single-cell immune profiling overview. (A) Schematic comparison of the CD7 *versus* CD19 CAR constructs, illustrating antigen-binding domain, hinge/transmembrane regions, and intracellular signaling modules. (B) Overview of the two cohorts (CD19 CAR-T for B-ALL and CD7 CAR-T for T-ALL/LBL), standardized blood collection timepoints (Pre, 14D, 28D, 2M, 3M), and analysis pipeline combining flow cytometry for circulating CAR detection with 40-marker CyTOF for high-dimensional immune profiling (pre-processing, clustering/annotation, and visualization). (C) Phenograph-defined clusters were annotated to major immune lineages; dot size denotes the percentage of positive cells within each cluster and dot color indicates scaled expression (Z-score) of the indicated markers. (D) Stacked bar charts show mean per-sample proportions of immune lineages across timepoints in CD19 *versus* CD7 recipients (including CD4^+^CAR^+^ T, CD8^+^CAR^+^ T, endogenous T cells, B cells, NK cells, monocytes, MDSCs, γδ T cells, and DCs). Sample sizes (CD19/CD7) at indicated time points: Baseline (*n* = 18/10), 14D (*n* = 17/9), 28D (*n* = 18/10), 2M (*n* = 10/5), and 3M (*n* = 4/6). (E) Uniform manifold approximation and projection (UMAP) plots of aggregated samples and distinct groups colored by annotated celltypes derived from CyTOF data. Sample sizes (CD19/CD7) at indicated time points: Baseline (*n* = 18/10), 14D (*n* = 17/9), 28D (*n* = 18/10), 2M (*n* = 10/5), and 3M (*n* = 4/6). MDSCs: myeloid-derived suppressor cells; CyTOF: cytometry by time-of-flight; CAR-T: chimeric antigen receptor T-cell.

**Table 2:** Baseline characteristics of the 28 population managed by CyTOF

Characteristic	Total (*N* = 28)	CD19 CAR-T (*N* = 18)	CD7 CAR-T (*N* = 10)	*P*-value
Gender, *n* (%)				0.705
Male	15 (53.6)	9 (50.0)	6 (60.0)	
Female	13 (46.4)	9 (50.0)	4 (40.0)	
Age				0.829
Median (years, interquartile range)	35 (26, 45)	35 (25, 48)	34 (30, 38)	
Disease, *n* (%)				<0.001
B-cell acute lymphoblastic leukemia	18 (64.3)	18 (100.0)	0 (0.0)	
T-cell acute lymphoblastic leukemia	10 (35.7)	0 (0.0)	10 (100.0)	
Bone marrow blast percentage, *n* (%)				0.705
<30%	15 (53.6)	9 (50.0)	6 (60.0)	
≥30%	13 (46.4)	9 (50.0)	4 (40.0)	
Pre_hematopoietic stem cell transplantation, *n* (%)				0.674
Yes	21 (75.0)	14 (77.8)	7 (70.0)	
No	7 (25.0)	4 (22.2)	3 (30.0)	
Response rate at day 28				>0.999
Complete response/Partial response	27 (96.4)	17 (94.4)	10 (100.0)	
No response	1 (3.6)	1 (5.6)	0 (0.0)	
Cytokine release syndrome, *n* (%)				0.682
0	6 (21.4)	5 (27.8)	1 (10.0)	
1	10 (35.7)	6 (33.3)	4 (40.0)	
2	12 (42.9)	7 (38.9)	5 (50.0)	
Immune effector cell–associated neurotoxicity syndrome, *n* (%)				>0.999
0	26 (92.9)	17 (94.4)	9 (90.0)	
1	2 (7.1)	1 (5.6)	1 (10.0)	
Early immune effector cell–associated hematotoxicity, *n* (%)				>0.999
0-2	6 (21.4)	4 (22.2)	2 (20.0)	
3-4	22 (78.6)	14 (77.8)	8 (80.0)	
Late immune effector cell–associated hematotoxicity, *n* (%)				0.016
0-2	15 (53.6)	13 (72.2)	2 (20.0)	
3-4	13 (46.4)	5 (27.8)	8 (80.0)	
Infection severity, *n* (%)				0.437
No	14 (50.0)	9 (50.0)	5 (50.0)	
Mild to severe	9 (32.1)	7 (38.9)	2 (20.0)	
Life-threatening to fatal	5 (17.9)	2 (11.1)	3 (30.0)	

CAR-T: chimeric antigen receptor T-cell; CD19 CAR-T: CD19-directed CAR-T therapy; CD7 CAR-T: CD7-directed CAR-T therapy; CyTOF: cytometry by time-of-flight.

### Global immune landscape of immune reconstitution following CAR-T therapy

Using UMAP for visualization and Phenograph for unsupervised clustering, we identified 49 distinct cell clusters. Guided by lineage-defining marker expression, these clusters were subsequently annotated into major immune lineages, including T cells (further resolved into CD4^+^, CD8^+^, and additional subsets), B cells, NK cells, monocytes, myeloid-derived suppressor cells (MDSCs), and dendritic cells (Figure 1C). The distribution of these annotated lineages across groups and timepoints is shown in the stacked composition plot (Figure 1D), while the integrated UMAP projection illustrates the global organization of immune populations (Figure 1E). Notably, phenotypic heterogeneity was preserved within each lineage, reflecting functional diversity. Subsequently, based on these annotation results, we systematically analyzed the abundance changes and functional states (*e.g*., activation and exhaustion) of different cell types.

Both CAR-T products effectively eliminated their respective target cells. CD19 CAR-T eradicated malignant and normal B cells, resulting in a declined immunoglobulin levels (IgG, IgA, and IgM) (Figure 2A and B), whereas CD7 CAR-T depleted both malignant and normal CD7^+^ T cells. Within the T-cell compartment, CD7^+^ T cells remained stable after CD19 CAR-T but declined sharply post-CD7 CAR-T, with incomplete recovery through Month 3 (Figure 2C and D). These lineage-specific effects created fundamentally different immune milieus in the two treatment cohorts.

**Figure 2:**
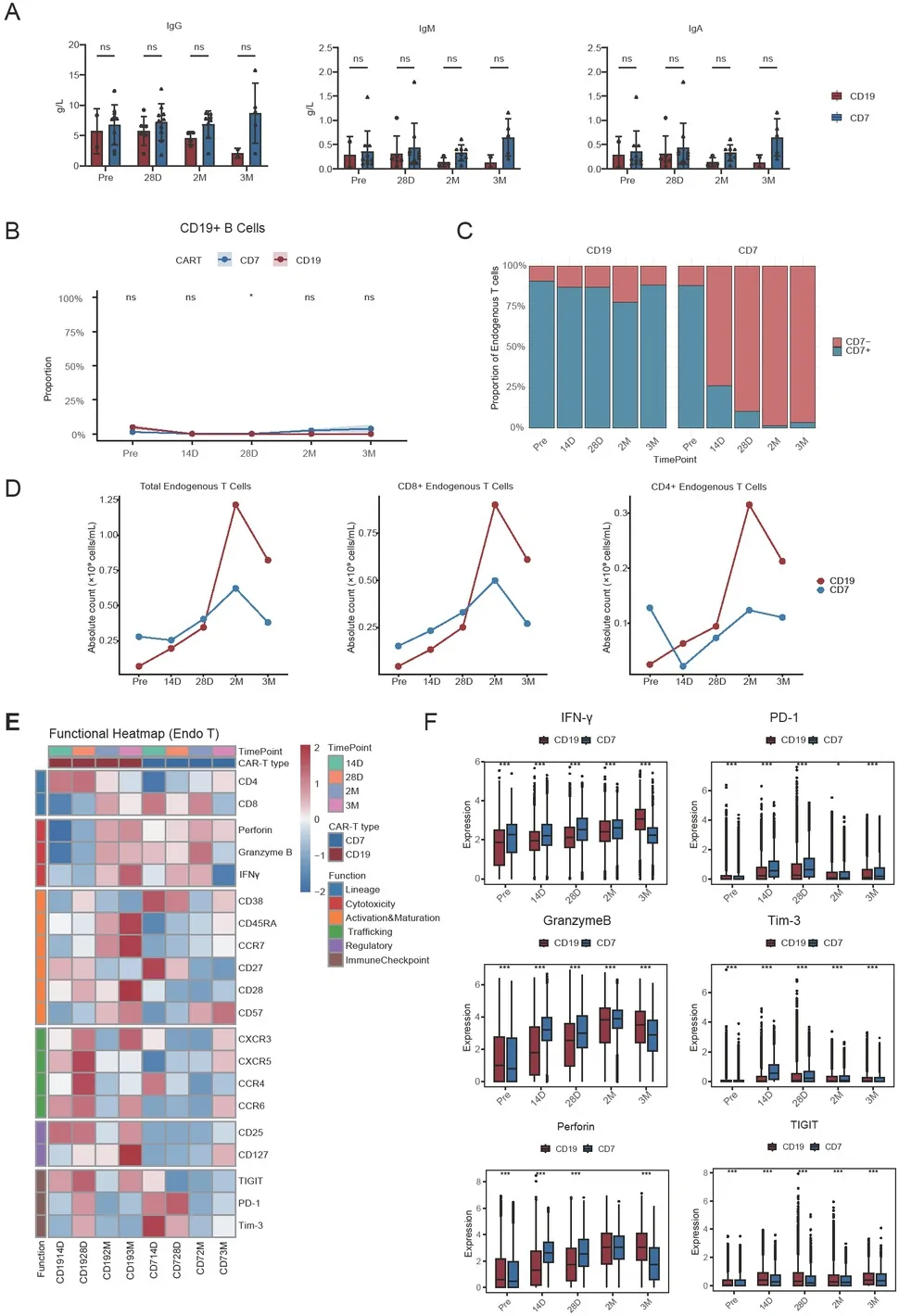
Target-cell depletion and remodeling of endogenous T-cell immunity. (A) Serum IgA, IgG, and IgM levels at matched timepoints. (B) Proportions of CD19^+^ B cells in peripheral blood over time comparing CD19 vs CD7 cohorts (individual values overlaid where shown). Sample sizes (CD19/CD7) at indicated time points: Baseline (*n* = 18/10), 14D (*n* = 17/9), 28D (*n* = 18/10), 2M (*n* = 10/5), and 3M (*n* = 4/6). (C) CD7 expression within endogenous T cells. Fraction of CD7^+^ cells among total endogenous T cells across timepoints by cohort. Sample sizes (CD19/CD7) at indicated time points: Baseline (*n* = 18/10), 14D (*n* = 17/9), 28D (*n* = 18/10), 2M (*n* = 10/5), and 3M (*n* = 4/6). (D) Absolute counts of endogenous T-cell subsets over time in CD19 and CD7 CAR-T recipients. Sample sizes (CD19/CD7) at indicated time points: Baseline (*n* = 18/10), 14D (*n* = 17/9), 28D (*n* = 18/10), 2M (*n* = 10/5), and 3M (*n* = 4/6). (E) CyTOF-derived scaled expression (Z-scores) of activation/maturation markers (CD27, CD28, CD38, CD57, CD45RA, CCR7), cytotoxic molecules (IFN-γ, granzyme B, perforin), trafficking receptors (CXCR3, CXCR5, CCR4, CCR6), regulatory receptors (IL-2Rα, IL-7Rα), and immune-checkpoint molecules (PD-1, TIM-3, TIGIT) by timepoint and cohort. (F) Boxplots of representative effector and checkpoint markers for endogenous T cells by cohort and timepoint. ^*^P < 0.05, ^***^P < 0.001; ns, not significant.

Beyond target compartments, a broader host immune environment also exhibited marked divergence. Endogenous T cells in the CD19 group gradually restoredcytotoxic capacity by Months 2–3, suggesting effective reconstitution. Conversely, the CD7 group exhibited transient cytotoxic peaks followed by waning co-stimulatory signaling and persistence, indicating premature exhaustion (Figure 2E and F).

Innate cells especially NK cells recovered robustly after CD19 CAR-T but poorly after CD7 CAR-T, reaching significantly higher levels at Day 28 and Month 3. Moreover, γδ T cells also showed delayed and incomplete rebound in the CD7 group at some time points after infusion (Figure 3A and B). While myeloid compartments imcluding monocytes, DCs and MDSCs remained comparable recovery patterns (Figure 3C). Collectively, these data reveal that CD7 CAR-T recipients experience deeper and more prolonged impairment across adaptive and innate compartments.

**Figure 3:**
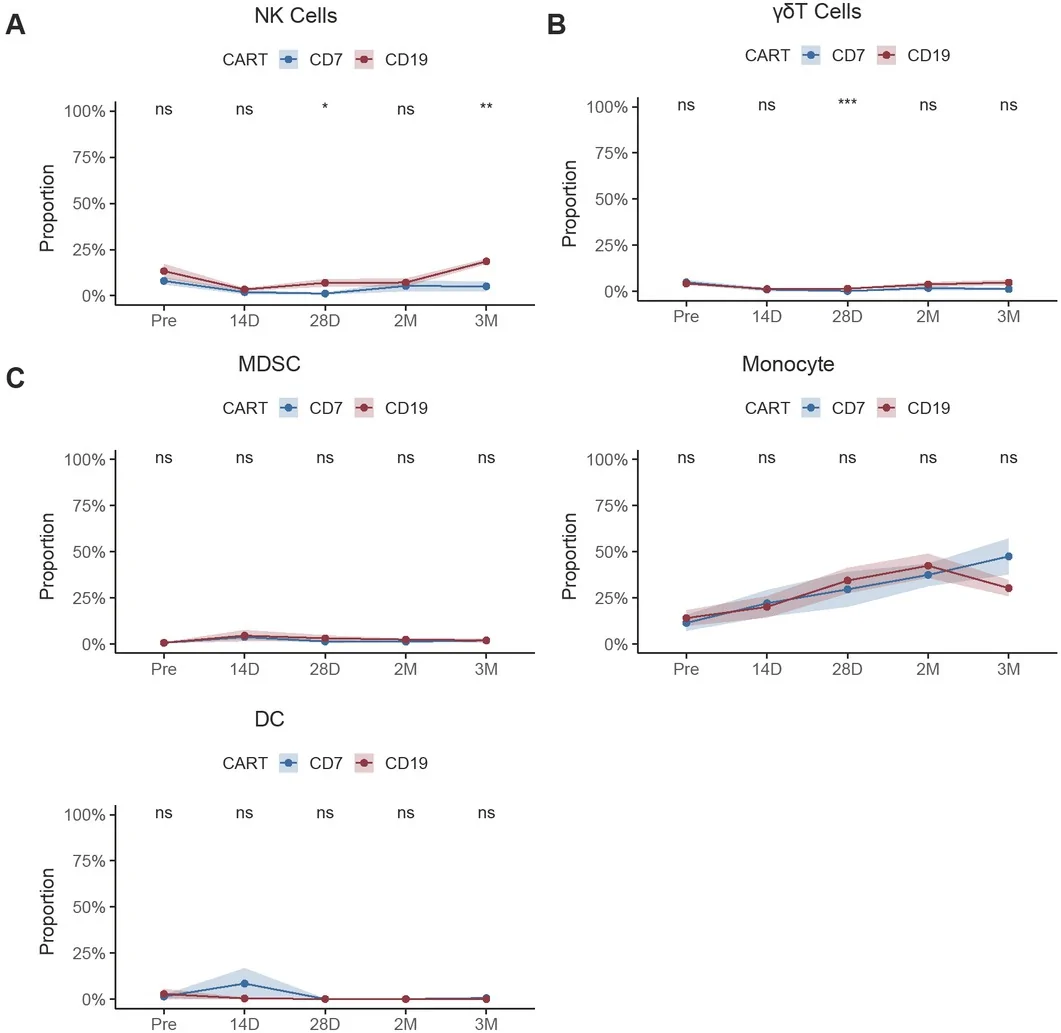
Reconstitution dynamics of other immune compartments after CAR-T therapy. (A) Recovery trajectories of NK cells over time after infusion in CD19 *versus* CD7 recipients. (B) Dynamics of γδ T-cell frequencies over time comparing CD19 and CD7 cohorts. (C) Temporal dynamics of other circulating innate cells frequencies in the two cohorts. Sample sizes (CD19/CD7) at indicated time points: Baseline (*n* = 18/10), 14D (*n* = 17/9), 28D (*n* = 18/10), 2M (*n* = 10/5), and 3M (*n* = 4/6). CAR-T: chimeric antigen receptor T-cell. ^*^P < 0.05, ^**^P < 0.01, ^***^P < 0.001; ns, not significant.

### CD7 CAR-T cells exhibit rapid activation followed by early and pronounced exhaustion compared with CD19 CAR-T cells

Further high-dimensional CyTOF analysis revealed profoundly distinct kinetic and functional profiles between the two CAR-T products. At Day 14, both CD7 and CD19 CAR-T cells exhibited early activation (elevated CD27/CD28) and high expression of cytotoxic molecules (IFN-γ, granzyme B, and perforin). Subsequently, CD7 CAR-T cells developed an exhaustion phenotype, characterized by sustained upregulation of co-inhibitory receptors (TIGIT, PD-1, and TIM-3), and was accompanied by a marked downregulation of cytotoxic molecules at late time points (Months 2 and 3). In contrast, CD19 CAR-T cells maintained a stable effector profile, sustained activation with stable effector function and low exhaustion markers, consistent with their long-term persistence (Figure 4A and B). To functionally validate these distinct *in vivo* trajectories, we subjected both CAR-T products to an *in vitro* repetitive target-cell stimulation assay. Consistent with our CyTOF findings, CD7 CAR-T cells exhibited a markedly accelerated exhaustion profile upon serial rechallenge. Compared to their CD19 counterparts, stimulated CD7 CAR-T cells expressed significantly higher levels of the co-inhibitory receptor PD-1 and demonstrated a more profound depletion of the cytotoxic molecule Granzyme B. While both products upregulated TIGIT and maintained comparable IFN-γ secretion following chronic exposure, the pronounced PD-1 elevation and Granzyme B loss in the CD7 cohort firmly corroborate their higher intrinsic propensity for rapid functional exhaustion (Figure 4C).

**Figure 4:**
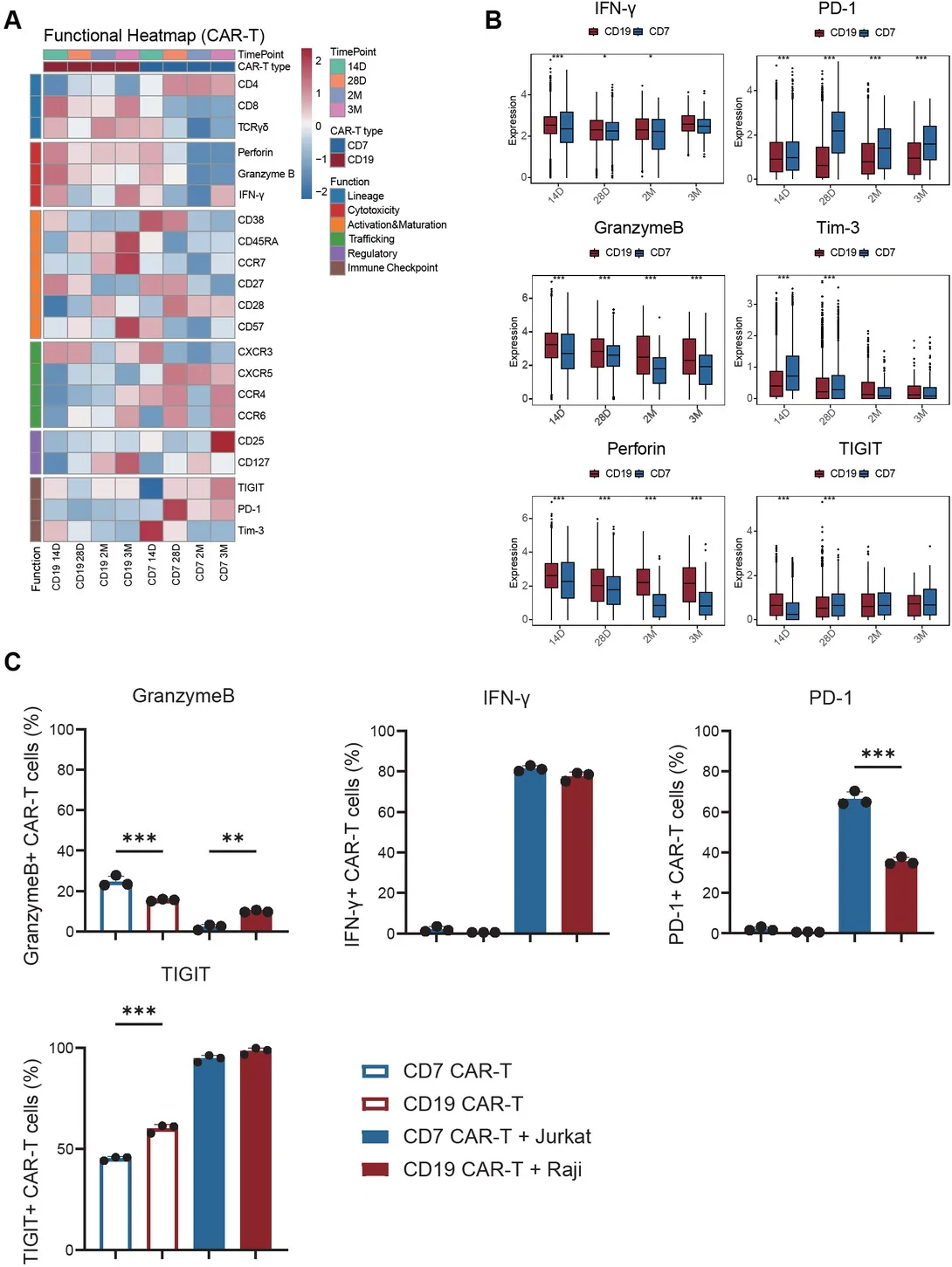
Functional phenotypes of CAR-T cells after CD7 *versus* CD19 CAR-T therapy. (A) CyTOF-derived scaled expression (Z-scores) of activation/maturation (CD27, CD28, CD38, CD57), cytotoxic effectors (IFN-γ, granzyme B, perforin), trafficking receptors (CXCR3, CXCR5, CCR4, CCR6), and exhaustion markers (PD-1, TIGIT, TIM-3) across timepoints and products. (B) Boxplots (by product and timepoint) for representative effectors (IFN-γ, granzyme B, perforin) and checkpoints (PD-1, TIGIT, TIM-3), illustrating early hyperactivation with rapid exhaustion in CD7 CAR-T *versus* steadier activation and lower exhaustion in CD19 CAR-T. (C) Expression of cytotoxic (Granzyme B, IFN-γ) and exhaustion (PD-1, TIGIT) markers in CD7 (blue) and CD19 (red) CAR-T cells at baseline (open bars) and following a 4-round repetitive target-cell stimulation (solid bars). Data are mean ± SD and analyzed by ANOVA with Tukey’s multiple comparisons test. ^*^P < 0.05, ^**^P < 0.01, ^***^P < 0.001. CyTOF: cytometry by time-of-flight; CAR-T: chimeric antigen receptor T-cell.

Chemokine receptor profiles further diverged that CD19 CAR-T cells expressed higher CXCR3, indicating stronger inflammatory homing, whereas CD7 CAR-T cells upregulated CXCR5, CCR4, and CCR6, suggesting follicular and mucosal tropism. These suggesting that CD7 CAR-T cells undergo rapid activation and tissue-homing differentiation but experience early functional decline.

We next examined product-intrinsic mechanisms of dysfunction by profiling activation and exhaustion features within the CAR-T compartment. A significantly higher frequency of CD38^+^HLA-DR^+^ CD8^+^ CAR-T cells was observed in the CD7 cohort compared with the CD19 cohort (Figure 5A), a phenotype associated with chronic activation and early functional decline in CAR-T products.^[29]^ Consistent with this profile, CD38^+^HLA-DR^+^ CD 8^+^ CAR-T cells showed strong positive correlations with PD-1^+^, TIGIT^+^, and TIM-3^+^ CAR-T populations (Figure 5B), indicating a tight association with an emerging exhaustion program. To functionally evaluate this specific subset under conditions mimicking chronic antigen exposure, we subjected the CAR-T infusion products to a 4-round repetitive target-cell stimulation assay (Figure 5C). Following this serial rechallenge, flow cytometric analysis revealed that the CD8^+^CD38^+^HLA-DR^+^ subset exhibited significantly higher expression of co-inhibitory receptors, specifically PD-1 and TIM-3, compared to the CD8^+^CD38^⁻^ HLA-DR^⁻^ population. Concurrently, this double-positive subset maintained significantly elevated levels of Granzyme B. This simultaneous expression of both a key cytotoxic molecule and exhaustion markers reflects a hyper-activated state that characteristically precedes functional exhaustion. These *in vitro* data align with our CyTOF observations, providing supportive evidence that the CD38^+^HLA-DR^+^CD8^+^ subset inherently possesses an exhaustion propensity under chronic stress. These findings suggest that CD7 CAR-T cells are more prone to exhaustion than CD19 CAR-T cells, potentially compromising their proliferative fitness and durability *in vivo*.

**Figure 5:**
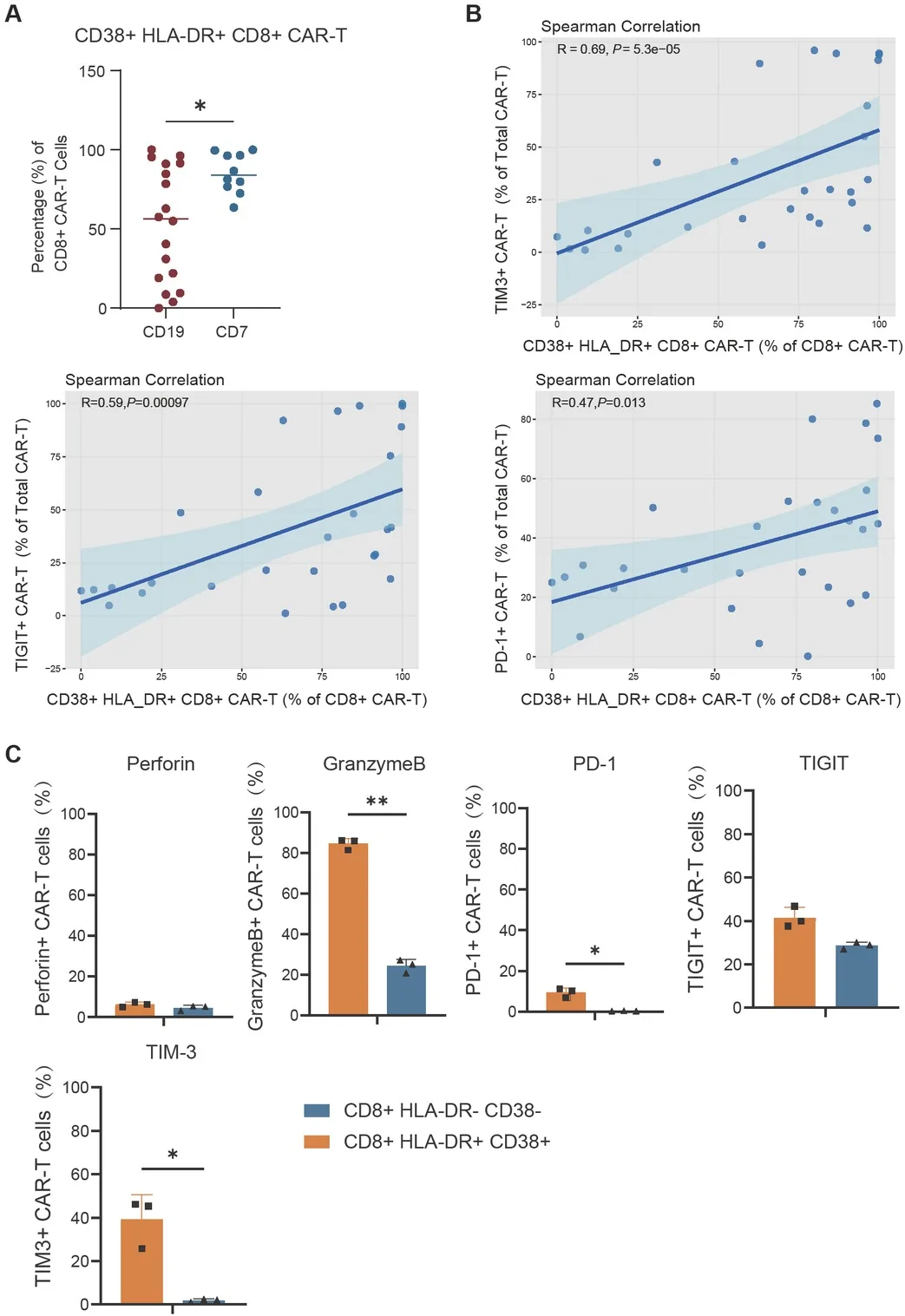
Activation and exhaustion characteristics of CD38^+^HLA-DR^+^ CD8^+^ CAR-T cells in patient samples and *in vitro*. (A) Proportion of CD38^+^HLA-DR^+^ CD8^+^ CAR-T cells within total CD8^+^ CAR-T cells in CD19 and CD7 cohorts at Day 28 post-infusion. (B) Positive associations between CD38^+^HLA-DR^+^ CD8^+^ CAR-T cells and exhausted CAR-T phenotypes (PD-1^+^, TIGIT^+^, and TIM-3^+^) at Day 28 (*n* = 28). (C) Flow cytometric analysis of cytotoxic (Perforin, Granzyme B) and exhaustion (PD-1, TIGIT, TIM-3) markers on CD8^+^CD38^–^HLA-DR^–^ (blue) and CD8^+^CD38^+^HLA-DR^+^ (orange) CAR-T cells following a 4-round repetitive target-cell stimulation assay. Data are shown as mean ± SD and analyzed by paired *t*-test. ^*^P < 0.05, ^**^P < 0.01. CAR-T: chimeric antigen receptor T-cell.

### CD19 CAR-T cells demonstrate superior longterm persistence compared with CD7 CAR-T cells

To characterize CAR-T expansion kinetics, we analyzed *in vivo* CAR-T cell levels in peripheral blood over time (Figure 6A). The median time to peak expansion was 14 days for both CD 19 CAR-T (range 10–28) and CD7 CAR-T (range 10–21).

**Figure 6:**
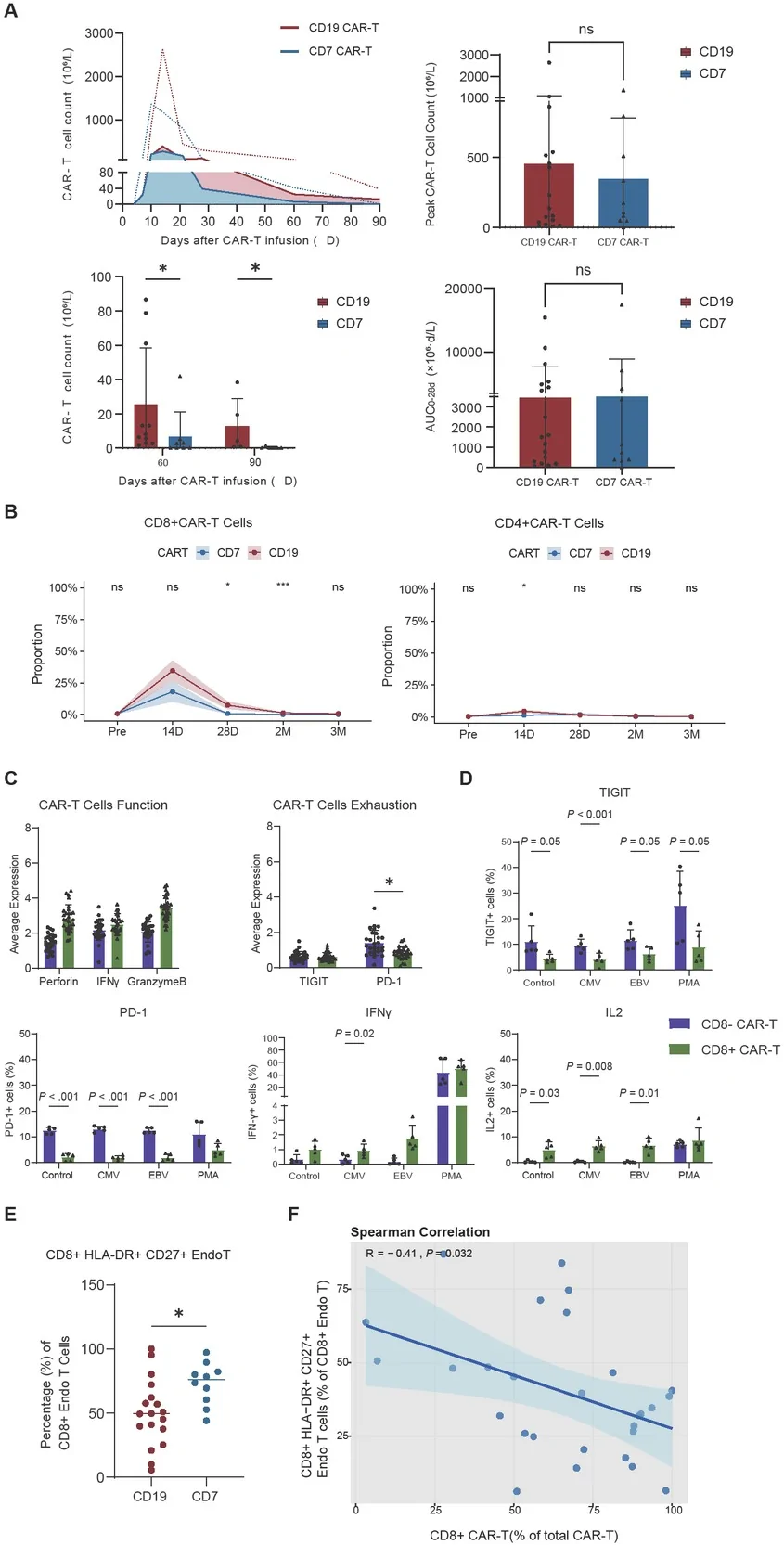
*In vivo* CAR-T kinetics, subset composition, and prognostic relevance. (A) Longitudinal curves of circulating CAR-T cells after infusion in the CD19 and CD7 cohorts; comparison of absolute CAR-T cell counts between the two groups at Days 60 and 90 post-infusion; comparisons of peak abundance (C_max) and AUC (0–28 d) between groups. AUC (0–28 d) denotes the area under the CAR-T expansion curve from Day 0 to Day 28; AUC (30–90 d) denotes the area under the CAR-T expansion curve from Day 30 to Day 90; bars indicate cohort means (individual values overlaid where shown). (B) Proportions of CD4^+^CAR^+^ and CD8^+^CAR^+^ T cells over time in each cohort, highlighting CD8^+^CAR^+^ dominance patterns (group means with variability bands). Sample sizes (CD19/CD7) at indicated time points: Baseline (*n* = 18/10), 14D (*n* = 17/9), 28D (*n* = 18/10), 2M (*n* = 10/5), and 3M (*n* = 4/6). (C) Functional and exhaustion markers of CD8^+^ and CD8- CAR-T cells *in vivo* (*n* = 28). (D) Comparison of function and exhaustion markers between CD8^+^ and CD8^–^ CAR-T cells after exogenous viral antigen peptide stimulation (*n* = 5). (E) Frequency of CD8^+^CD27^+^HLA-DR^+^ cells in CD19 *versus* CD7 CAR-T products at Day 28 (*n* = 28). (F) Negative correlation between CD27^+^HLA-DR^+^CD8^+^ cells and CD8^+^ CAR-T-cell proportion (*n* = 28). CAR-T: chimeric antigen receptor T-cell. ^*^P < 0.05,^***^P < 0.001; ns, not significant.

While both products expanded to similar peak magnitudes (C_max_: 182.5 × 106/L for CD19 *vs*. 141.0 × 106/L for CD7, *P* = 0.94), their subsequent kinetic profiles diverged substantially. The cumulative exposure within the first 28 days post-infusion (AUC_0–28_ d) was comparable between CD7 and CD19 CAR-T cells (934.1 *vs*. 1548 × 10^6^ cells·days/L, *P* = 0.80). However, absolute CAR-T cell counts remained higher in the CD 19 group at both Month 2 and Month 3, suggesting superior long-term persistence.

### CD8^+^ CAR-T cell dominance shapes functional fitness and clinical outcomes

At day 28, the proportion of CD8^+^ CAR^+^ T cells was significantly higher in CD19 CAR-T recipients compared with CD7 CAR-T (*P* = 0.0028, Figure 6B). We compared the functional profiles of CD 8^+^ and CD8^⁻^ CAR-T cells. *In vivo*, CD8^+^ CAR-T cells showed stronger effector activity, with higher expression of perforin, IFN-γ, and granzyme B than CD8^⁻^ CAR-T cells (Figure 6C). Conversely, PD-1 expression was substantially lower in CD8+ CAR-T cells, indicating a less exhausted effector state.

To assess these differences under antigen-specific conditions, we stimulated CD8^+^ and CD8^⁻^ CAR-T subsets with CMV- and EBV-derived peptide pools (Figure 6D). After stimulation, perforin levels were comparable between the two subsets, whereas CD8^+^ CAR-T cells exhibited markedly higher induction of IFN-γ and IL-2, reflecting stronger activation and greater polyfunctionality. Exhaustion markers showed the opposite trend. CD8^⁻^ CAR-T cells exhibited more pronounced PD-1 upregulation after antigen stimulation, whereas CD8^+^ CAR-T cells showed only minimal increases. TIGIT displayed a similar pattern: CD8^⁻^ CAR-T cells demonstrated robust induction following stimulation, while CD8^+^ CAR-T cells showed lower TIGIT upregulation.

Notably, a distinct endogenous CD8^+^HLA-DR^+^CD27^+^ subset was markedly enriched in the CD7 group (Figure 6E). This population has recently been defined as a regulatory precursor-like subset with intrinsic immunosuppressive function capable of suppressing T cell proliferation and activation, both *in vitro* and *in vivo* by our group.^[30]^ Consistent with this biology, correlation analysis revealed that the frequency of CD8^+^HLA-DR^+^CD27^+^ cells was negatively associated with the proportion of functional CD8^+^ CAR-T cells (Figure 6F), suggesting that this regulatory-like population may restrain CD8^+^ CAR-T expansion.

### Inferior clinical outcomes associated with CD7 CAR-T therapy compared with CD19 CAR-T therapy

Given the marked differences in immune reconstitution between the two CAR-T products, particularly the deeper and more prolonged immunosuppression following CD7

CAR-T, we anticipated that such biological disparities would translate into distinct clinical outcomes. All 74 patients achieved high initial responses (overall response rate 90.5%), with comparable day-28 CR rates between CD19 and CD7 CAR-T (88.5% *vs*. 90.9%, *P* = 0.999). However, CD7 CAR-T recipients showed a trend toward higher early relapse within 6 months (31.8% *vs*. 14.7%, *P* = 0.080) and experienced significantly worse long-term outcomes, including higher 1-year NRM (32.4% *vs*. 9.2%, *P* = 0.017), lower 1-year disease-free survival (31.8% *vs*. 54.2%, *P* = 0.030), and inferior overall survival (44.6% *vs*. 78.8%, *P* = 0.013) (Figure 7A–D). We futher conducted univariate and multivariate analysis to identified potential risk factors including HSCT history, baseline bone marrow blast, base line immune status (ANC count), and previous treatment lines for NRM. Variables with *P* < 0.1 in univariate analysis were entered into a multivariate Cox regression model. As presented in Supplementary Table S2, the CAR-T group was the only independent risk factor associated with NRM.

**Figure 7:**
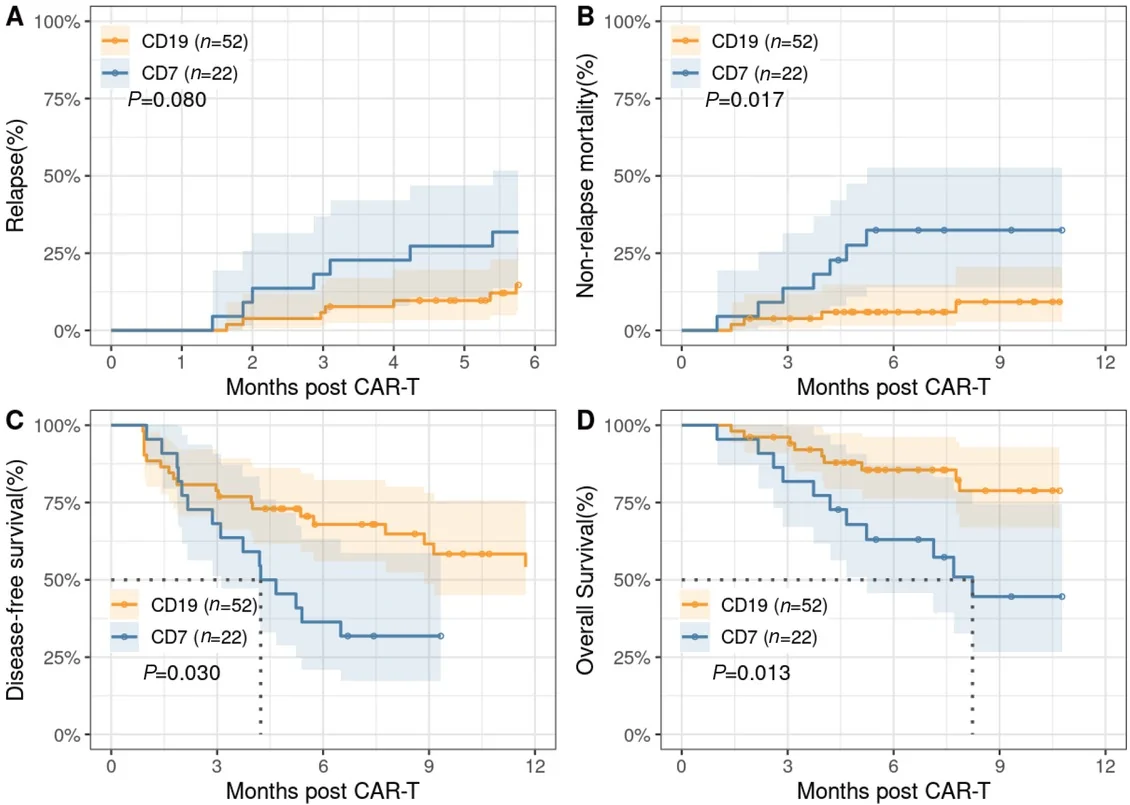
Clinical outcomes after CD19 *vs*. CD7 CAR-T therapy. (A) Cumulative incidence of relapse. (B) NRM. (C) DFS. (D) OS. Curves compare CD19 (*n* = 52) and CD7 (*n* = 22) cohorts up to 12 months post-infusion; shaded bands show 95% CIs and P values are from log-rank tests (as indicated on each panel). CD7 CAR-T shows higher 1-year NRM and inferior DFS/OS despite similar early responses. CAR-T: chimeric antigen receptor T-cell; NRM: non-relapse mortality; DFS: disease-free survival; OS: overall survival.

As a higher NRM rate were observed in CD7 group, we futher analysis hematologic toxicity and infectious complications. Although the incidence of early ICAHT was similar between groups, CD7 CAR-T recipients developed significantly higher rates of grade 3 to 4 late ICAHT within 3 months post-infusion (59.1% *vs*. 30.8%, *P* = 0.023). This prolonged immunesuppression was associated with a higher lifetheratening infections in the CD7 CAR-T cohort. Life-threatening to fatal infection occurred in 31.8% 7/22) of CD7 patients, compared to 5.8% (3/52, *P* = 0.009) in the CD19 group (Table 1).

In summary, our clinical data showed that while both CD19 and CD7 CAR-T therapies induced high initial response rates, CD7 CAR-T treatment was associated with significantly worse long-term survival outcomes, higher non-relapse mortality, increased late hematologic toxicity, and a greater burden of severe infectious complications.

## Discussion

In this study, we applied high-dimensional CyTOF with a 40-marker panel to longitudinally profile immune dynamics after CD19 and CD7 CAR-T therapy. By integrating the phenotypic data and clinical outcomes, we identified markedly distinct immune trajectories between the two treatment settings associated with differences in CAR-T cell persistence, immune reconstitution, and non-relapse mortality. Importantly, given the intrinsic biological differences between B-ALL and T-ALL/LBL, these findings should be interpreted as disease context specific rather than purely target driven.

Previous studies focused on differences in CAR design and manufacturing, such as those observed between various CD19 or BCMA CAR-T products, which can influence expansion, durability, and toxicity; however, the impact of targeting fundamentally different immune lineages on post-infusion immune remodeling remains elusive. Our data highlight the fundamentally different post-infusion dynamics of CD19 and CD7 CAR-T cells. CD19 CAR T cells exhibit sustained activation and a progressive differentiation trajectory, accompanied by durable target cell clearance and relatively preserved endogenous T-cell immunity. This balanced immune remodeling likely contributes to lower late toxicity and reduced NRM. In contrast, CD7 CAR-T cells display rapid expansion followed by early functional exhaustion, characterized by persistent upregulation of inhibitory receptors (PD-1, TIGIT, and TIM-3) and loss of effector capacity. To corroborate these *in vivo* observations, we subjected both CD19 and CD7 CAR-T cells to an *in vitro* repetitive target-cell stimulation assay. Consistent with our clinical data, while both CAR-T products maintained comparable cytokine secretion (IFN-γ), CD7 CAR-T cells exhibited a more profound depletion of cytotoxic capacity and a more rapid acquisition of an exhausted phenotype compared to CD19 CAR-T cells upon serial rechallenge.

The clinical implications of these divergent immune dynamics are underscored by their associations with adverse outcomes. In the CD7 cohort, early hyperactivation followed by exhaustion of CD7 CAR-T cells was associated with severe hematological toxicity, delayed immune recovery, and markedly increased rates of fatal infections, contributing to higher NRM. These observations align with real-world evidence in B-NHL, showing that impaired immune recovery predicts infection, disease progression, and mortality.^[31]^ Similarly, studies comparing different anti-BCMA CAR-T products have revealed that specific phenotypic signatures correlate with differences in expansion, toxicity, and infection risk, reinforcing the notion that product-specific kinetics imprint long-term clinical outcomes.^[16]^

Given these divergent immune trajectories, we next examined whether common phenotypic determinants of CAR-T cell fitness could explain the variability in persistence and clinical outcomes between the two products. Among these determinants, CD8^+^ CAR-T cells have been consistently correlated with sustained function across different CAR-T platforms. Samara *et al*. reported that a CD 8^+^ CAR-T expansion ratio > 1.87 predicted significantly longer progression-free survival (PFS) and overall survival (OS).^[32]^ In BCMA CAR-T therapy, Ho *et al*. showed that a CD4/CD8 ratio > 1 on day 7 was strongly correlated with delayed immune-related toxicity and increased non-relapse mortality.^[33]^ Similarly, Galli *et al*. demonstrated that lower CD4/CD8 ratios in infused CD 19 CAR-T products were associated with improved responses, further emphasizing the relevance of CD8^+^-skewed CAR-T products.^[34]^ Consistent with these observations, we found that CD7 CAR-T recipients exhibited lower CD8^+^ CAR-T cell proportions, which were associated with poorer immune recovery and higher NRM. In exploratory analyses, a higher proportion of CD8^+^ CAR-T cells at day 28 was associated with lower 1-year NRM, particularly in the CD7 cohort (Supplementary Figure S1). However, given the limited sample size and lack of validation, this finding should be interpreted with caution as it does not support a definitive threshold for clinical use. Rather, these data suggest that CD 8^+^ enrichment may reflect more favorable CAR-T cell persistence and immune reconstitution, warranting further investigation in larger cohorts.

In addition to the observed differences in CAR-T kinetics and immune reconstitution, we identified an expansion of CD8^+^HLA-DR^+^CD27^+^ regulatory T cells and accumulation of the CD8^+^ CD38^+^HLA-DR^+^ CAR-T subset in the CD7 cohort. As comprehensively characterized in our previous work, the CD8^+^HLA-DR^+^CD27^+^ subset exerts potent suppression on T-cell proliferation and effector functions, a capacity we have rigorously validated through both *in vitro* assays and *in vivo* humanized mouse models.^[30]^ Its inverse association with CD8+ cell abundance suggests that this regulatory-like population may directly limit the clonal expansion of CAR-T cells. In addition, CD7 CAR-T recipients show persistent accumulation of CD38^+^HLA-DR^+^ CAR-T cells, a phenotype associated with metabolic restriction and exhaustion program enforcement.^[29]^ Importantly, our *in vitro* functional assays corroborated these phenotypic associations under chronic antigen exposure conditions. Following repetitive target-cell stimulation, the CD8^+^CD38^+^HLA-DR^+^ subset exhibited significantly higher expression of co-inhibitory receptors (PD-1 and TIM-3) while maintaining elevated levels of Granzyme B. The simultaneous expression of cytotoxic molecules and exhaustion markers implied that this subset was driven into a hyperactivated state, which characteristically precipitated rapid functional exhaustion under chronic stress. Although these findings provide a plausible cellular basis for the early dysfunction observed *in vivo*, extrapolating *in vitro* stress responses directly to the complex clinical CAR-T microenvironment remains speculative, and elucidating the precise *in-vivo* triggers of this specific phenotype awaits future investigation.

Our CyTOF-based immune mapping provides a high-resolution view of these processes, enabling the correlation of early CAR-T cell activation and exhaustion states with downstream complications such as immune effector cell-associated hematotoxicity,^[25,35,36]^ opportunistic infections, and relapse. These findings highlight that target antigen selection shapes not only antitumor efficacy, but also the depth and duration of post-infusion immunosuppression, underscoring the need for antigen-specific supportive strategies such as optimized infection prophylaxis and immune monitoring in patients receiving CD7^-^directed therapy.

This study has some limitations. First, the sample size, particularly for longitudinal CyTOF monitoring in the CD7 CAR-T cohort was limited, which may reduce the statistical power and affect generalizability. Second, comparisons between CD19 CAR-T in B-ALL and CD7 CAR-T in T-ALL/LBL are inherently confounded by differences in disease biology; thus, the observed differences in immune dynamics may partly reflect distinct baseline immune contexts rather than purely target-specific effects. Third, this study is primarily descriptive and based on phenotypic associations; therefore, it cannot definitively establish causality between the observed immune phenotypes and clinical outcomes without further functional validation. In addition, the limited sample availability precludes additional mechanistic experiments. Finally, long-term follow-ups and validation in larger multicenter cohorts with integrated functional analyses are essential to confirm and extend these findings.

In conclusion, this study revealed that CD7 CAR-T therapy is constrained by rapid functional decline and inadequate immune recovery, leading to inferior survival outcomes compared with those of CD19 therapy. These findings support the need for improved CD7 CAR-T design and tailored immune monitoring strategies to mitigate toxicity and improve outcomes in T-cell malignancies.

## Supplementary Information

Supplementary materials are only available at the official site of the journal (www.intern-med.com).

## Supplementary Material

Supplementary Material Details
