# Supplementary material for: Distinct cellular dynamics following CD19 and CD7 CAR-T therapies revealed by high-dimensional CyTOF analysis and impact on clinical outcomes
Source: J Transl Int Med. 2026 Oct 23;14(5):822–37. doi: 10.1515/jtim-2026-0081 (PMC13726658; doi:10.1515/jtim-2026-0081)
Supplement: Supplementary file 1 [file jtim-2026-0081_sm.pdf]

Supplementary materials

Supplementary Table S1. A 40-marker CyTOF antibody panel of this study.

Fcs_colname	Antigen
Bi209Di	CD11b
Dy161Di	CD196_CCR6
Dy162Di	CD123_IL_3Ra
Dy163Di	CD4
Dy164Di	CD185_CXCR5
Er166Di	CD183_CXCR3
Er167Di	CD8
Er168Di	CD57
Er170Di	CD127_IL_7Ra
Eu151Di	CD33
Eu153Di	CD27
Gd155Di	CD45RA
Gd156Di	CD28
Gd157Di	Perforin
Gd158Di	CD197_CCR7
Gd160Di	CAR
Ho165Di	CD66b
In115Di	CD3
La139Di	IFN_g
Lu175Di	CD16
Nd142Di	TCR_g_d
Nd143Di	Granzyme_B

Nd144Di	CD14
Nd145Di	TIGIT
Nd146Di	CD5
Nd148Di	CD19
Nd150Di	CD7
Pr141Di	CD56
Pt195Di	CD34
Sm147Di	CD15
Sm149Di	CD25_IL_2Ra
Sm152Di	IgD
Sm154Di	CD24
Tb159Di	CD11c
Tm169Di	CD45
Yb171Di	CD279_PD_1
Yb172Di	CD38
Yb173Di	CD366_Tim_3
Yb174Di	CD194_CCR4
Yb176Di	HLA_DR

Supplementary Table S2. Univariate and multivariate analyses of risk factors associated with non-relapse mortality.

Variable	Univariate Analysis		Multivariate Analysis	
	**HR (95% CI)**	***P* Value**	**HR (95% CI)**	***P* Value**
CAR-T Group	3.223 (1.936-11.093)	**0.063**	3.984 (1.239-12.814)	**0.020**
Patient's age	1.944 (0.518-7.299)	0.325		
Patient's gender	1.106 (0.342-3.570)	0.866		
Base line bone marrow blast	1.033 (0.204-5.235)	0.538		
HSCT History	1.573 (0.371-6.665)	0.980		
Base line immune status	0.983 (0.259-3.725)	0.969		
Previous treatment lines	1.931 (0.500-7.462)	0.340		

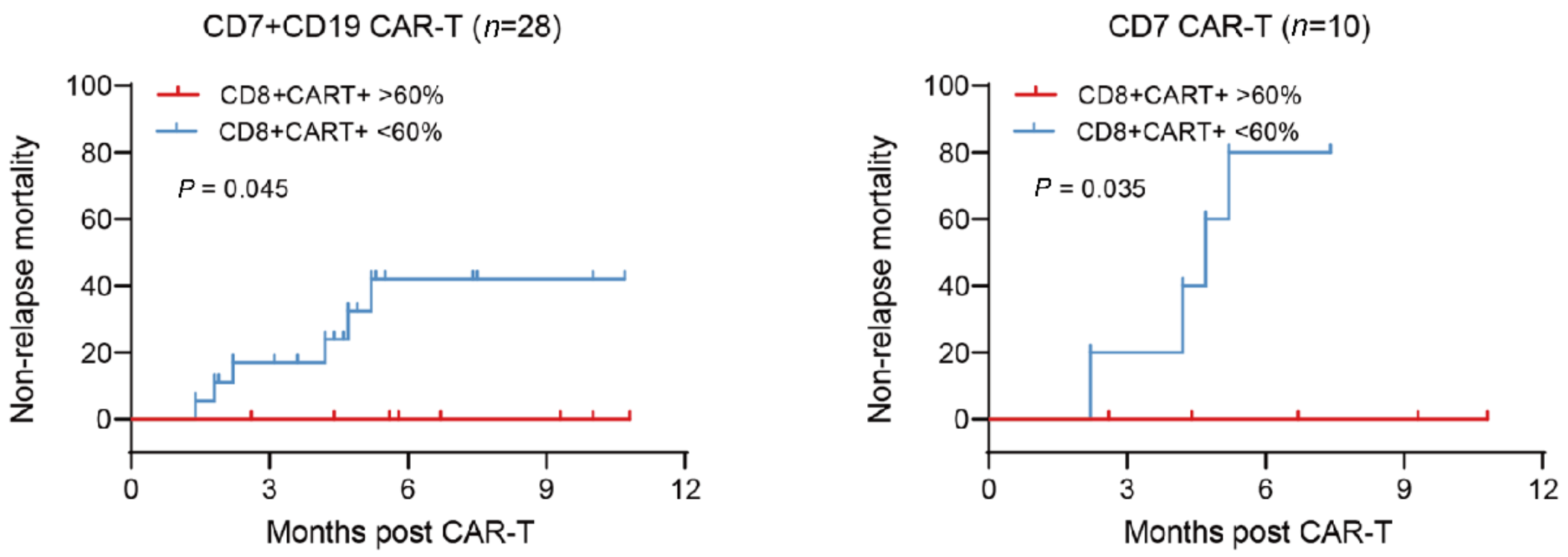

Supplementary Figure S1. $CD8^{+}$ CAR-T ratio ⩾ 60% on Day 28 was associated with a lower NRM. (A) Kaplan–Meier curves for non-relapse mortality (NRM) stratified by Day-28 $CD8^{+}CAR^{+}$ fraction ⩾ 60% *vs.* < 60% (overall monitored cohort, with CD7-specific analysis where applicable).
